# Integrated BLV surveillance in Kazakhstan, 2025: diagnostic complementarity and risk zoning

**DOI:** 10.3389/fvets.2026.1798232

**Published:** 2026-03-13

**Authors:** Saltanat Mamanova, Markhabat Kassenov, Ainur Nurpeisova, Saira Kaimoldina, Elvira Bashenova, Aiken Karabassova, Vladimir Kirpichenko, Perizat Akshalova, Fariza Ikramkulova, Aigul Kassen, Bakhyt Tulepov, Marat Turkeyev, Zhandos Abay, Maksat Serikov, Zhibek Zhetpisbay, Malik Yussupov, Nurlan Akhmetsadykov, Tolganay Imanbekova, Kunsulu Zakarya, Nurkuisa Rametov, Raikhan Nissanova

**Affiliations:** 1 Kazakh Scientific Research Veterinary Institute, LLP (KazSRVI), Almaty, Kazakhstan; 2 Department of Computer Science, Kazakh National University, Almaty, Kazakhstan; 3 Research and Production Enterprise Antigen LLP, Almaty, Kazakhstan; 4 National Holding Qazbiopharm, JSC, Astana, Kazakhstan; 5 Tecton Analytics LLP, Astana, Kazakhstan

**Keywords:** BLV, bovine leukemia virus, diagnostic complementarity, Kazakhstan, PCR, seroprevalence, spatial analysis, surveillance

## Abstract

**Background:**

Bovine leukemia virus (BLV) is a cell-associated retrovirus that remains endemic in many cattle-producing countries. In the absence of effective vaccination, BLV control relies on sustained surveillance and accurate interpretation of serological and molecular diagnostic data. However, the systematic integration of multiple diagnostic layers with spatial analysis at the national level remains limited in endemic settings.

**Methods:**

In 2025, a nationwide integrated surveillance of BLV was conducted in the Republic of Kazakhstan. A total of 3,650 serum samples were collected from 140 epidemiological units across 17 administrative regions. Cattle aged ≥24 months were examined using agar gel immunodiffusion (AGID) and enzyme-linked immunosorbent assay (ELISA) for serological screening, and polymerase chain reaction (PCR) for detection of proviral DNA. Partial sequencing was performed on selected PCR-positive samples for viral confirmation. Spatial analysis and territorial risk zoning were carried out using Geographic Information System (GIS)–based visualization to assess regional distribution patterns.

**Results:**

BLV infection exhibited pronounced spatial heterogeneity across the country. Serological screening identified regions with varying levels of BLV circulation, while PCR confirmed proviral presence in seropositive animals and in a limited number of seronegative cases. Discrepancies between AGID, ELISA, and PCR results reflected differences in diagnostic sensitivity and infection stage rather than methodological inconsistency. Integration of serological, molecular, and spatial data enabled classification of territories into distinct risk zones and identification of localized clusters of increased BLV circulation.

**Conclusion:**

Integrated surveillance combining serological testing, molecular detection, and spatial analysis provides a robust framework for assessing BLV epidemiology in endemic countries. The 2025 data from Kazakhstan demonstrate that diagnostic complementarity is essential for accurate spatial risk zoning and epidemiological interpretation. This framework supports risk-based decision-making and is readily transferable to other BLV-endemic settings with heterogeneous production systems, potentially informing the development of targeted surveillance and control strategies.

## 1 Introduction

Bovine leukemia virus (BLV) is a cell-associated deltaretrovirus that causes enzootic bovine leukosis, a chronic lymphoproliferative disease of cattle (1, 2). The infection is characterized by lifelong persistence of proviral DNA integrated into the host genome, predominantly in B lymphocytes (3, 4). Infected animals usually remain clinically asymptomatic for prolonged periods, while a subset develops persistent lymphocytosis or, less frequently, lymphosarcoma (5, 6). The absence of efficient viral clearance and the lack of a commercially available vaccine make BLV control dependent on surveillance, management practices, and removal or segregation of infected animals (7, 8).

BLV transmission occurs primarily through the transfer of infected lymphocytes, which distinguishes it from many other viral infections of livestock (9). Consequently, virus circulation is closely associated with iatrogenic factors, animal management practices, and vertical transmission rather than environmental persistence or aerosol spread (10). These biological characteristics result in heterogeneous patterns of infection at both herd and regional levels, particularly in countries where cattle production systems vary widely and biosecurity measures are inconsistently applied (11, 12).

Serological testing remains the cornerstone of BLV surveillance worldwide. Agar gel immunodiffusion (AGID) and enzyme-linked immunosorbent assay (ELISA) are internationally recognized methods for detecting antibodies against BLV structural proteins and are routinely used for population-level screening (13, 14). While ELISA provides high analytical sensitivity and suitability for large-scale testing, AGID continues to be applied in endemic regions due to its simplicity, low infrastructure requirements, and regulatory acceptance. However, serological methods reflect host immune response rather than direct viral presence and may yield discordant results depending on the stage of infection (15).

Molecular detection of proviral DNA using polymerase chain reaction (PCR) complements serological testing by enabling direct identification of infected animals (16), including those in early stages of infection or with atypical antibody responses (17). PCR-based methods also support confirmatory diagnostics and epidemiological investigations, while sequencing of selected genomic regions allows verification of viral identity and contributes to molecular epidemiology (18–20). Despite these advantages, molecular tools are often applied selectively rather than systematically in national surveillance programs, limiting their integration into routine epidemiological assessment (17).

Spatial analysis has increasingly been recognized as an important component of infectious disease surveillance in livestock populations. Geographic visualization of diagnostic results facilitates the identification of regional heterogeneity, localized clusters, and areas of epidemiological stability. In the context of BLV, where infection dynamics are influenced by management practices and animal movement, spatial risk zoning provides a practical framework for prioritizing control measures and allocating resources (21).

Kazakhstan represents a typical endemic setting for BLV, characterized by diverse cattle production systems, regional variability in veterinary infrastructure, and ongoing animal movements between territories. Although serological monitoring is routinely performed, integrated analyses combining serological, molecular, and spatial data remain limited. As a result, interpretation of surveillance results and risk assessment at the national level remain fragmented.

The objective of this study was to conduct an integrated nationwide surveillance of BLV in Kazakhstan in 2025, combining serological testing, molecular detection, and spatial analysis. By evaluating diagnostic complementarity and applying risk-based spatial zoning, this study aims to provide a comprehensive assessment of BLV epidemiology and to support evidence-based approaches to surveillance and control in endemic settings.

## 2 Materials and methods

### 2.1 Study design and surveillance framework

An integrated nationwide surveillance study of BLV was conducted in the Republic of Kazakhstan in 2025. The study was designed as a cross-sectional epidemiological survey integrating serological, molecular, and spatial data to assess the national distribution of BLV and to evaluate diagnostic complementarity under endemic conditions. Sampling and laboratory testing were carried out within the framework of routine veterinary surveillance programs and targeted epidemiological investigations implemented at the national level. Sampling was conducted within the framework of the official state veterinary surveillance program operating across all 17 administrative regions of the Republic of Kazakhstan. The surveillance system follows a stratified administrative structure in which regional veterinary authorities implement routine monitoring plans covering registered epidemiological units (farms or livestock holdings). Herd (epidemiological unit) selection was based on regional surveillance schedules and field logistics within the routine monitoring plan rather than formal probabilistic random sampling. Sampling within epidemiological units was performed by authorized field veterinarians during routine herd handling. Animals were selected to reflect herd structure (production category/age groups) and this procedure represents operational surveillance practice rather than a formally randomized within-herd sampling scheme. Within each selected epidemiological unit, cattle aged ≥24 months were sampled according to standard surveillance practice, prioritizing animals representative of the herd structure and production category rather than clinically suspected individuals. The number of samples collected per region reflected regional surveillance quotas and livestock distribution, providing nationwide geographic coverage. Because sampling followed the routine surveillance plan and regional quotas, sampling intensity varied across regions. Therefore, national estimates should be interpreted as surveillance-based indicators with nationwide geographic coverage rather than as design-based estimates from a probability sample.

### 2.2 Study population and sample collection

Cattle aged ≥24 months were included in the study, as this age group is considered the most informative for BLV surveillance due to the presence of established serological responses. A total of 3,650 serum samples were collected from cattle aged ≥24 months from 140 epidemiological units across 17 administrative regions of Kazakhstan. Animals originated from multiple administrative regions of Kazakhstan, representing diverse cattle production systems and epidemiological settings.

Blood samples were collected by authorized veterinary personnel in accordance with national veterinary regulations. Serum samples were obtained for serological testing, and whole-blood samples were collected for molecular analysis. Sample collection did not involve experimental infection or procedures beyond routine veterinary practice.

### 2.3 Serological testing

Serological screening for BLV antibodies was performed using AGID and a competitive ELISA. In 2025, a total of 3,650 serum samples were collected in 140 epidemiological units across 17 administrative regions of the Republic of Kazakhstan. AGID was conducted to detect antibodies against the BLV envelope glycoprotein gp51 using the commercial kit IDVET BLV (IDvet, Grabels, France). The method is based on radial diffusion of antigen and antibodies in agar gel; formation of a visible precipitation line at the antigen–antibody interface was interpreted as a positive result. Results were read visually and instrumentally in accordance with the manufacturer’s instructions. In parallel, sera were tested by competitive ELISA using ID Screen® BLV Competition (IDvet, Grabels, France) for detection of antibodies targeting BLV gp51. The assay is based on competitive binding of serum antibodies to viral antigens immobilized on microplate wells, followed by enzymatic signal detection. This approach was applied to improve detection sensitivity, including at early stages of infection.

### 2.4 Molecular detection

Molecular detection of BLV proviral DNA was performed on 1,527 whole-blood samples collected during the same surveillance period. Genomic DNA was extracted using the QIAamp DNeasy Blood & Tissue Kit (QIAGEN, Hilden, Germany) according to the manufacturer’s protocol, including cell lysis and purification on silica spin columns.

Real-time PCR (qPCR) was performed using a pooling strategy and the QuantiTect Multiplex PCR NoROX Kit (QIAGEN, Hilden, Germany) to amplify a BLV proviral target using the following oligonucleotides: MRF (forward; positions 2,321–2,339) 5′-CCTCA ATTCCTTTAAACTA-3′, MRR (reverse; positions 2,421–2,440) 5′-GTACCGGGAAGACTGGATTA-3′, and MRBLV probe (positions 2,340–2,360) 6FAM-GAACGCCTCCAGGCCCTTCA-BHQ1.

Samples were combined into pools consisting of five individual whole-blood DNA extracts per pool. In the event of a positive amplification signal in a pooled sample, all individual samples included in the pool were subsequently tested separately to identify BLV-positive animals. A sample was considered positive when a specific amplification curve with a characteristic exponential profile was observed under the predefined cycling conditions, while the absence of amplification in negative controls was used to confirm assay specificity.

Thermal cycling conditions were as follows: polymerase activation at 96 °C for 10 min, followed by 37 cycles of denaturation at 94 °C for 45 s, annealing at 58 °C for 1 min, and extension at 72 °C for 1 min, with a final extension at 72 °C for 7 min. qPCR results were interpreted based on detection of the specific amplified proviral fragment in tested pools and corresponding individual samples.

For molecular characterization of circulating BLV strains, genomic DNA was additionally isolated from peripheral blood leukocytes of seropositive animals and subjected to nested PCR targeting the env gene, yielding a final amplicon of 444 bp.

### 2.5 Sequencing and genetic confirmation

Nested PCR amplification of the BLV env gene was carried out in two stages using Evrogen 5 × Screen Mix (Evrogen, Moscow, Russia). First-round PCR employed primers env5032/env5608 to generate a 600 bp product under the following conditions: initial denaturation at 94 °C for 2 min; 39 cycles of 95 °C for 30 s, 62 °C for 30 s, and 72 °C for 60 s; followed by a final extension at 72 °C for 4 min. Second-round PCR used primers env5099/env5521 to generate a 444 bp product with the following cycling parameters: initial denaturation at 94 °C for 2 min; 39 cycles of 95 °C for 30 s, 70 °C for 30 s, and 72 °C for 60 s; and a final extension at 72 °C for 4 min.

Amplicons were visualized by electrophoresis in 1.5% agarose gel with ethidium bromide intercalation. PCR products intended for sequencing were purified using the GeneJET PCR Purification Kit (Thermo Scientific, Vilnius, Lithuania). Sanger sequencing was performed using the BigDye Terminator v3.1 Cycle Sequencing Kit (Thermo Fisher Scientific, Waltham, MA, United States) with cycling parameters of 96 °C for 20 s, 50 °C for 10 s, and 60 °C for 4 min (cycle sequencing). Post-reaction cleanup was conducted using the BigDye XTerminator Purification Kit (Thermo Fisher Scientific, Waltham, MA, United States), and sequencing was performed on a GA3500 Genetic Analyzer (Applied Biosystems, Foster City, CA, United States) in accordance with the manufacturer’s instructions.

Sequence comparison and phylogenetic analyses were conducted using MEGA version 11 (Pennsylvania State University, University Park, PA, United States). Phylogenetic trees were constructed using the neighbor-joining method with bootstrap analysis based on 1,000 replicates. Bootstrap values ≥50% are shown at branch nodes.

### 2.6 Spatial analysis and risk zoning

Geographic Information System (GIS)–based spatial analysis was performed to visualize the distribution of BLV-positive cases across the Republic of Kazakhstan in 2025 and to support territorial risk zoning. Spatial analyses were conducted using ArcMap version 10.8 (ESRI, Redlands, CA, United States), together with auxiliary analytical tools GARP and GeoDa. Vector-based administrative maps of the Republic of Kazakhstan at a scale of 1:1,000,000 were used as the cartographic basis (Kazakhstan Center for Geoinformation Systems LLP).

Spatial data layers were prepared and processed using ArcView GIS, ArcGIS Spatial Analyst, and ArcGIS Geostatistical Analyst (ESRI, Redlands, CA, United States). These tools were applied for data integration, spatial aggregation, and visualization of epidemiological indicators at the level of administrative territories and epidemiological units.

Based on integrated serological and molecular diagnostic results, administrative territories were classified into low-, moderate-, and high-risk zones reflecting different levels of BLV circulation. Risk-zoning criteria (prespecified). Regions were classified using predefined thresholds based on ELISA seroprevalence (primary indicator): high risk ≥20%, moderate risk 5–<20%, and low risk <5%. To account for regional uncertainty, classifications were interpreted alongside the Wilson 95% confidence intervals. PCR testing was performed on a selected subset of samples; therefore, PCR results were used as confirmatory evidence of BLV circulation (presence/absence) and did not contribute to prevalence estimation. As a robustness check, zoning based on serology-only thresholds was compared with the integrated serology+PCR classification to identify any changes attributable to molecular confirmation.

Spatial visualization was used to identify regional heterogeneity and localized clustering of BLV-positive cases; no predictive modeling or spatiotemporal forecasting was performed. The spatial assessment was primarily descriptive (GIS-based visualization), and formal spatial autocorrelation/cluster statistics (e.g., Moran’s I, Getis–Ord Gi, SaTScan) were not applied in this study.

### 2.7 Statistical analysis

Statistical analysis was conducted with consideration of the binomial nature of the data, characterized by discrete outcomes (positive/negative) and heterogeneous sample sizes. Because standard methods based on normal approximation may lead to biased estimates under conditions of low event frequencies or extreme proportions, a set of specialized statistical procedures was applied to ensure analytical validity. Disease prevalence and corresponding 95% confidence intervals were estimated using the Wilson score interval, which provides superior coverage accuracy compared with the Wald interval and reduces bias in small samples and at boundary values (22). All confidence intervals were calculated at the animal level using Wilson score intervals, assuming independence. No adjustment for within-herd clustering (e.g., cluster-robust or design-effect–based approaches) was applied; therefore, confidence intervals may be overly narrow in the presence of strong herd-level correlation.

Regional prevalence estimates were compared with the overall incidence level using the proportions Z-test when sample sizes were sufficient, whereas the exact binomial test was employed in cases with limited numbers of positive observations or zero counts to maintain robustness under rare-event conditions (23). To determine whether the observed differences reflected random variation or a systematic effect, Fisher’s exact test was applied; this test, based on the hypergeometric distribution, yields exact probability estimates and is particularly suitable for small samples and sparse contingency tables (24). Inter-method agreement between AGID and ELISA was assessed using Cohen’s Kappa coefficient, quantifying concordance beyond chance (25). The standard error of Kappa was used to construct 95% confidence intervals, allowing evaluation of estimate precision and discrimination between true agreement and sampling variability. Additionally, McNemar’s test was applied to account for the paired nature of the data, specifically to evaluate marginal homogeneity and determine whether the observed diagnostic shift between AGID and ELISA represented a systematic detection bias rather than stochastic variation (26). This analytical approach ensured statistical rigor and reliable interpretation of the results. Because animals were sampled within epidemiological units, potential clustering at the herd level cannot be entirely excluded; therefore, prevalence estimates are interpreted as surveillance-based indicators with broad geographic coverage rather than as design-based estimates from a probability sample.

### 2.8 Ethical considerations

This study was conducted within the framework of official veterinary surveillance programs of the Republic of Kazakhstan. Sample collection was performed by authorized veterinary personnel as part of routine diagnostic and monitoring activities and did not involve experimental infection, animal experimentation, or invasive procedures beyond standard veterinary practice. Therefore, according to national regulations, no additional approval from an institutional animal ethics committee was required.

## 3 Results

### 3.1 Overall serological surveillance results (2025)

In 2025, nationwide serological surveillance for BLV was conducted across the Republic of Kazakhstan. A total of *N* = 3,650 cattle aged ≥24 months were examined using serological methods. BLV antibodies were detected in 609 animals, corresponding to an overall ELISA-based seroprevalence of 16.68% at the national level. Using AGID, 274 seropositive animals were identified, yielding an overall AGID seroprevalence of 7.50%. Seropositive animals were detected in multiple administrative regions, whereas several regions remained seronegative throughout the study period. Marked regional heterogeneity in BLV seroprevalence was observed. ELISA-based seroprevalence ranged from 0.00 to 41.01%, while AGID-based seroprevalence varied between 0.00 and 22.33% across regions.

Table 1 summarizes the regional distribution of serological testing results obtained by AGID and ELISA. Regions with the highest ELISA seroprevalence included Jetisu Region (41.01%) and Kyzylorda Region (40.33%), whereas no seropositive animals were detected in several regions, including Atyrau, Abai, Aktobe, and Pavlodar Regions by either method.

**Table 1:** Overall serological surveillance results for BLV in Kazakhstan, 2025.

No.	Region	Planned number of samples	Tested by AGID	AGID-positive samples	AGID seroprevalence, %	Tested by ELISA	ELISA-positive samples	ELISA seroprevalence, %
1	Jetisu	434	434	70	16.12	434	178	41.01
2	Kyzylorda	300	300	67	22.33	300	121	40.33
3	Akmola	171	171	30	17.54	171	66	38.59
4	Kostanay	240	240	21	8.75	240	57	23.75
5	West Kazakhstan	260	260	15	5.76	260	47	18.07
6	North Kazakhstan	390	390	36	9.23	390	65	16.66
7	East Kazakhstan	135	135	3	2.22	135	11	8.14
8	Mangystau	88	88	5	5.68	88	7	7.95
9	Karaganda	235	235	8	3.40	235	16	6.80
10	Zhambyl	200	200	8	4.00	200	12	6.00
11	Almaty	320	320	5	1.56	320	17	5.31
12	Ulytau	221	221	5	2.26	221	8	3.62
13	Turkestan	295	295	1	0.33	295	4	1.35
14	Atyrau	47	47	0	0.00	47	0	0.00
15	Abai	135	135	0	0.00	135	0	0.00
16	Aktobe	89	89	0	0.00	89	0	0.00
17	Pavlodar	90	90	0	0.00	90	0	0.00
	Total	3,650	3,650	274	7.50	3,650	609	16.68

### 3.2 Comparison of AGID and ELISA results

Both AGID and ELISA were applied for serological testing in 2025. The majority of samples showed concordant results between the two methods. However, discordant outcomes were recorded, including ELISA-positive/AGID-negative and AGID-positive/ELISA-negative cases.

AGID-positive results were predominantly observed in territories with established BLV circulation. The distribution of concordant and discordant results varied across regions.

Analysis of serological data demonstrated that the overall incidence of bovine infection in the Republic of Kazakhstan in 2025 was 7.51% (95% CI, 6.70–8.41) as determined by AGID, whereas a substantially higher incidence was detected by ELISA, reaching 16.68% (95% CI, 15.51–17.93).

At the regional level, AGID-based incidence was absent or minimal in Atyrau, Abai, Aktobe, Pavlodar, and Turkestan regions (0–0.3%), with confidence intervals overlapping zero and no statistically significant deviation from the national average according to the proportions Z-test, exact binomial test, or Fisher’s exact test (*p* ≈ 1.0). Low to moderate AGID incidence was observed in Almaty, West Kazakhstan, Ulytau, Kostanay, and Mangystau regions (1.5–8.7%); although these values did not differ significantly from the pooled national level in Z-test and binomial analyses (*p* > 0.05), Fisher’s exact test indicated localized heterogeneity in the distribution of positive cases (*p* < 0.05).

In contrast, significantly elevated AGID incidence was recorded in Kyzylorda (22.3%), Zhetysu (16.1%), and Akmola (17.5%) regions, where all applied statistical tests consistently demonstrated a significant excess relative to the national mean.

Regional ELISA estimates and their statistical comparisons are summarized in Table 2.

**Table 2:** Comparison of AGID and ELISA results for BLV detection in cattle, Kazakhstan, 2025.

No	Region	AGID					ELISA					Cohen’s Kappa with SE	McNemar’s test *p*-value
		Incidence rate, %	95% CI (Wilson)	*p*-value (Z-test)	*p*-value (Binom.)	*p*-value (Fisher)	Incidence rate, %	95% CI (Wilson)	*p*-value (Z-тест)	*p*-value (Binom.)	*p*-value (Fisher)		
1	Akmola	17.5	[12.57, 23.94]	0.0003	<0.0001	<0.0001	38.6	[31.63, 46.07]	<0.0001	<0.0001	<0.0001	0.506 ± 0.0636	<0.001
2	Almaty	1.5	[0.67, 3.60]	1.0000	1.0000	0.0308	5.3	[3.34, 8.34]	1.0000	1.0000	<0.0001	0.441 ± 0.1313	0.0015
3	Jetisu	16.1	[12.97, 19.88]	<0.0001	<0.0001	<0.0001	41.0	[36.48, 45.70]	<0.0001	<0.0001	<0.0001	0.433 ± 0.0389	<0.001
4	Atyrau	0.0	[0.00, 7.56]	1.0000	1.0000	1.0000	0.0	[0.00, 7.56]	1.0000	1.0000	1.0000	-	-
5	East Kazakhstan	2.2	[0.76, 6.33]	1.0000	0.9981	0.1236	8.1	[4.61, 14.00]	0.9999	0.9987	0.0004	0.408 ± 0.1636	0.0133
6	Abai	0.0	[0.00, 2.77]	1.0000	1.0000	1.0000	0.0	[0.00, 2.77]	1.0000	1.0000	1.0000	-	-
7	Zhambyl	4.0	[2.04, 7.69]	0.9943	0.9851	0.0036	6.0	[3.47, 10.19]	1.0000	1.0000	0,0002	0.790 ± 0.1017	0.134
8	Kostanay	8.7	[5.79, 13.01]	0.2478	0.2643	<0.0001	23.7	[18.81, 29.52]	0.0051	0.0031	<0.0001	0.471 ± 0.0690	<0.001
9	Aktobe	0.0	[0.00, 4.14]	1.0000	1.0000	1.0000	0.0	[0.00, 4.14]	1.0000	1.0000	1.0000	-	-
10	West Kazakhstan	5.7	[3.53, 9.30]	0.8853	0.8845	<0.0001	18.1	[13.88, 23.21]	0.2799	0,2,973	<0.0001	0.434 ± 0.0772	<0.001
11	Karaganda	3.4	[1.73, 6.57]	0.9997	0.9972	0.0037	6.8	[4.23, 10.77]	1.0000	1.0000	<0.0001	0.651 ± 0.1137	0.0133
12	Ulytau	2.2	[0.97, 5.19]	1.0000	0.9998	0.0305	3.6	[1.85, 6.98]	1.0000	1.0000	0,0037	0.763 ± 0.1322	0.248
13	Kyzylorda	22.3	[17.99, 27.38]	<0.0001	<0.0001	<0.0001	40.3	[34.94, 45.97]	<0.0001	<0.0001	<0.0001	0.597 ± 0.0455	<0.001
14	Mangystau	5.6	[2.45, 12.62]	0.7702	0.7988	0.0295	7.9	[3.91, 15.52]	0.9988	0.9945	0.0069	0.822 ± 0.1228	0.48
15	Pavlodar	0.0	[0.00, 4.09]	1.0000	1.0000	1.0000	0.0	[0.00, 4.09]	1.0000	1.0000	1.0000	-	-
16	North Kazakhstan	9.2	[6.74, 12.51]	0.1198	0.1177	<0.0001	16.6	[13.30, 20.69]	0.5039	0.5249	<0.0001	0.674 ± 0.0550	<0.001
17	Turkestan	0.3	[0.06, 1.89]	1.0000	1.0000	0.5000	1.3	[0.53, 3.43]	1.0000	1.0000	0.0619	0.397 ± 0.2764	0.248
Total		7.51	[6.70, 8.41]	-	-	-	16.68	[15.51, 17.93]	-	-	-	0.5714 ± 0.02	<0.001

Inter-method agreement, quantified using Cohen’s Kappa coefficient, ranged from moderate to substantial across regions, reflecting variable concordance between AGID and ELISA results. Regions with higher Kappa values exhibited stronger agreement, indicating consistent detection of positive cases by both methods. Conversely, lower *κ* values suggest that ELISA identified additional infections undetected by AGID, highlighting the method’s higher diagnostic sensitivity and the potential underestimation of regional prevalence when relying solely on AGID. To evaluate the statistical significance of this detection bias, McNemar’s test was performed. The analysis revealed a highly significant systematic divergence between the two assays at the national level (χ^2^ = 333.00, *p* < 0.001). At the regional level, the shift in diagnostic yield was most pronounced in Jetisu (χ^2^ = 106.01, *p* < 0.001), Kyzylorda (χ^2^ = 52.02, *p* < 0.001), Akmola, and Kostanay (both χ^2^ = 34.03, *p* < 0.001), where ELISA identified a significantly higher number of positive samples that were missed by AGID. In regions with very low prevalence or small sample sizes, such as Mangystau and Ulytau, the difference did not reach statistical significance (*p* > 0.05). Overall, McNemar’s test confirmed a significant asymmetry of discordant AGID/ELISA pairs at the national level and in several high-prevalence regions.

**Figure 1:**
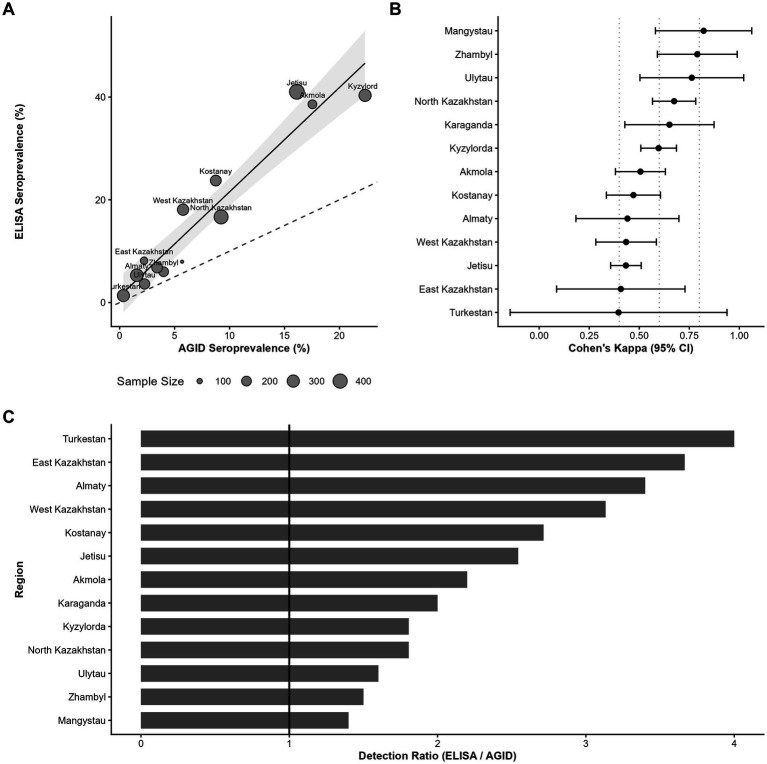
Regional comparison of disease prevalence estimated by AGID and ELISA: **(A)** Systematic sensitivity shift relative to identity reference; **(B)** Agreement beyond chance quantified by Cohen’s Kappa; **(C)** Detection gap indicating relative diagnostic underestimation.

Figure 1 provides a graphical summary of the regional estimates reported in Tables 1, 2 to facilitate interpretation of between-assay differences, whereas the tables are retained to present exact numerical values for reproducibility and secondary analyses. To objectively compare the diagnostic performance of AGID and ELISA, a multi-dimensional analytical framework was implemented in Figure 1. A correlation analysis with a 1:1 identity line was used to visualize the systematic diagnostic shift and the between-assay detection shift relative to the 1:1 line (Figure 1A). The statistical robustness of inter-method agreement was evaluated using a forest plot of Cohen’s Kappa with 95% confidence intervals (CI), benchmarking regional results against standardized agreement scales (Figure 1B). Furthermore, a detection gap between AGID and ELISA was calculated to quantify the fold-increase in positive case identification, identifying regional disparities in the underestimation of seroprevalence by traditional methods (Figure 1C).

### 3.3 Molecular detection of BLV proviral DNA

PCR targeting BLV proviral DNA was performed on a selected subset of whole-blood samples to complement serological findings and to assess diagnostic concordance. From the total collection of 1,527 whole-blood samples, 550 samples were included in PCR analysis. This subset comprised all available seropositive animals identified by AGID and/or ELISA, as well as a limited number of seronegative samples selected for diagnostic clarification, in order to detect potential early or atypical infections and to evaluate diagnostic complementarity between serological and molecular methods.

BLV proviral DNA was detected in 60 animals, corresponding to an overall PCR positivity rate of 10.9% among the tested subset. PCR-positive animals were identified in multiple administrative regions, confirming BLV infection at the molecular level across geographically distinct areas. No exclusive clustering of PCR-positive cases restricted to a single region was observed; instead, molecularly confirmed infections were detected in territories characterized by varying levels of serological prevalence.

Regional PCR positivity rates varied substantially (Table 3). The highest proportion of PCR-positive samples was observed in the East Kazakhstan Region (34.07%), followed by Mangystau (12.50%), West Kazakhstan (9.09%), Karaganda (6.25%), Almaty (4.44%), and Zhetisu (4.44%) Regions. No PCR-positive samples were detected in Kostanay, Kyzylorda, Ulytau, Aktobe, Pavlodar, and Zhambyl Regions during the study period.

**Table 3:** PCR results in relation to serological status of cattle examined in 2025.

Region	Number of samples	PCR-positive samples	PCR positivity, %	Farms with positive results
Almaty	90	4	4.44	Farm “Amiran” (1), Farm Abdulliev (1), LLP “Rakhat” (1), LLP village Bolek (1)
Kostanay	80	0	0.00	–
West Kazakhstan	66	6	9.09	Farm “Kanagat” (2), Farm “Akzhol” (1), LLP “Dostyk–Konyss” (1), Farm “Imran” (2)
Jetisu	45	2	4.44	Farm Shyakhmetov (1), Farm Moldakhmetov (1)
Kyzylorda	20	0	0.00	–
Ulytau	22	0	0.00	–
East Kazakhstan	135	46	34.07	Private household (v. Kalzhyr – 7), Private household (v. Soldatovo – 8), Private household (v. Altynbel – 9), Private household (v. Kokbastau – 9), Private household (v. Solovyevo – 2), Private household (v. Maleevskoe – 5), Private household (v. Nikolsk – 5), Private household (v. Markakol – 1)
Karaganda	16	1	6.25	LLP “Global Beef Products” (1)
Aktobe	11	0	0.00	–
Mangystau	8	1	12.50	Private household, Zhanaozen (1)
Pavlodar	9	0	0.00	–
Zhambyl	30	0	0.00	–
Total	550	60	10.90	–

PCR-positive cases were identified across different farm types, including commercial farms, limited liability partnerships, and private households, indicating heterogeneous patterns of BLV circulation at the local level. The detection of proviral DNA in both seropositive animals and a limited number of diagnostically selected seronegative cases supports the complementary role of molecular testing within an integrated BLV surveillance framework.

### 3.4 Sequencing and genetic confirmation

Partial sequencing was performed on selected PCR-positive samples (*n* = 5) to confirm viral identity. All obtained nucleotide sequences were identified as BLV and showed high similarity to reference BLV sequences available in public databases. No unexpected genetic divergence or non-BLV sequences were detected within the analyzed fragments.

**Figure 2:**
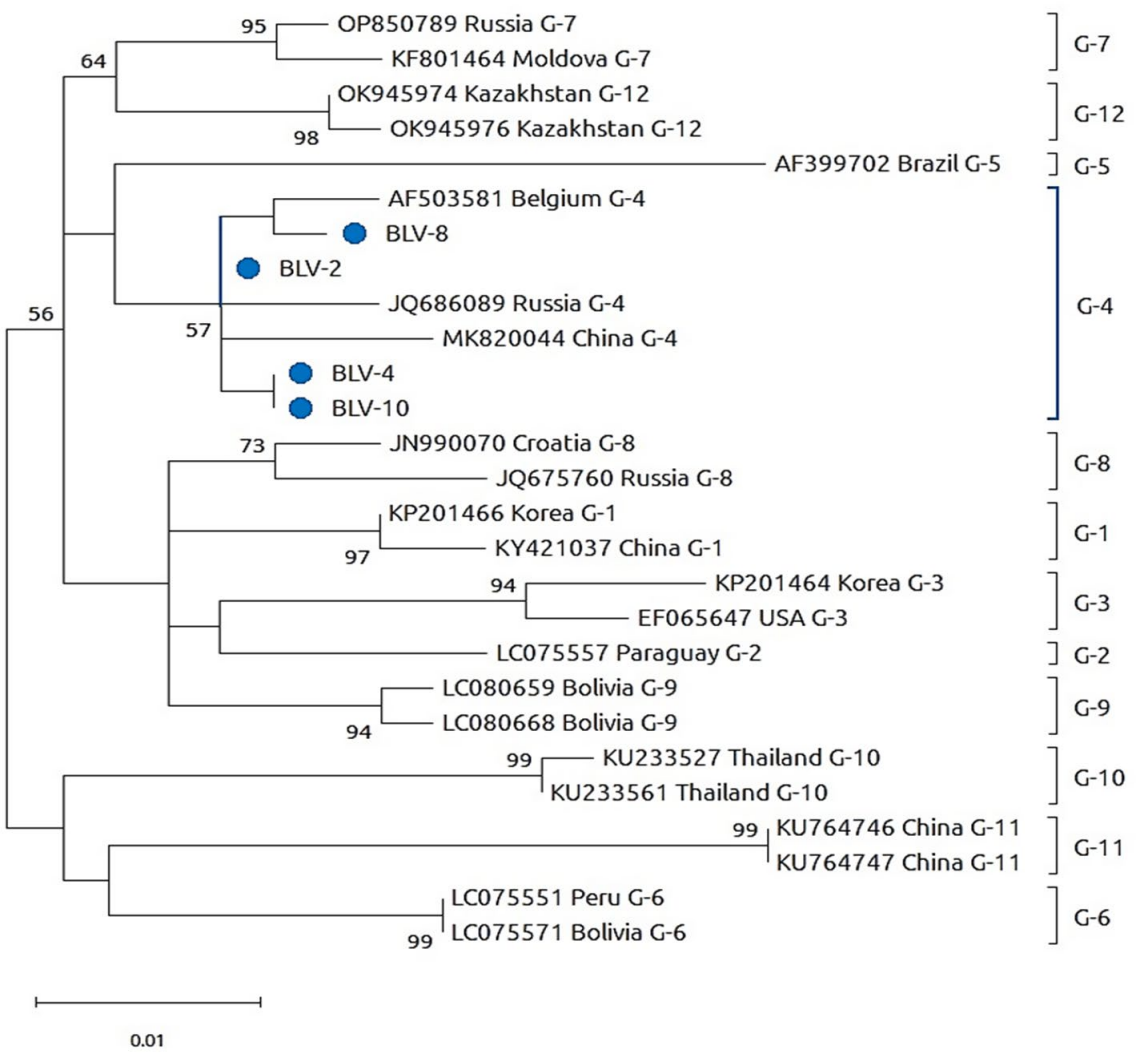
Genetic confirmation of BLV by partial sequencing.

Sequencing was conducted exclusively for molecular confirmation purposes and was not intended to support comprehensive phylogenetic reconstruction. The analyzed sequences clustered within established BLV genotypes and showed close relatedness to previously reported strains from neighboring and geographically connected regions (Figure 2; Table 4).

**Table 4:** BLV-positive samples identified by PCR.

No.	Region, district	Settlement/farm	Sample ID	Ct value
1	Almaty Region, Talgar District	Talgar town, Farm “Amiran”	No. 10	22.79
2	East Kazakhstan Region, Zaysan District	Zhylyptinsky rural district, Farm “Kanagat”	No. 4	26.25
3	Jetisu Region, Karatal District	Zholbarys rural district, Farm Shyakhmetov	No. 2	21.19
4	East Kazakhstan Region, Markakol District	Markakol rural district, private household	No. 14	22.65
5	Karaganda Region, Aktogay District	Kezhek village, LLP “Global Beef Products”	No. 8	23.06

Phylogenetic analysis of partial BLV genomic sequences obtained from PCR-positive cattle samples collected in Kazakhstan in 2025. The tree was constructed based on partial env gene sequences using the neighbor-joining method with bootstrap analysis (1,000 replicates). Bootstrap values ≥50% are shown at branch nodes. Sequences generated in this study are indicated by blue circles and labeled as BLV-2, BLV-4, BLV-8, and BLV-10. Reference BLV sequences representing major genotypes (G-1 to G-12) were retrieved from public databases and included for comparative analysis. The letter “G” denotes BLV genotype classification. The scale bar represents 0.01 nucleotide substitutions per site.

As shown in Figure 2, BLV sequences obtained from samples No. 2 and No. 10 demonstrated 100% nucleotide identity with BLV strains previously reported in Russia, indicating a recent common source or introduction through regional cattle movements. In addition, samples No. 4 and No. 10 exhibited 99.8–100% nucleotide similarity to BLV strains reported from Mongolia (2014) and Russia (2014), corresponding to the globally distributed G-4 genotype. The geographic origin of the sequenced samples, including Almaty, East Kazakhstan, Jetisu, and Karaganda Regions, overlaps with major livestock movement corridors and breeding material logistics routes, providing a biologically plausible explanation for the observed genetic similarity with regional reference strains. Cycle threshold (Ct) values of the sequenced samples ranged from 21.19 to 26.25, indicating a relatively high proviral load in the analyzed animals and supporting their epidemiological relevance in ongoing BLV transmission. The nucleotide sequences generated in this study were deposited in the GenBank database under the following accession numbers: PX367239 (BLV_env_KZ_Sample02), PX367240 (BLV_env_KZ_Sample04), PX367241 (BLV_env_KZ_Sample08), and PX367242 (BLV_env_KZ_Sample10).

### 3.5 Spatial distribution of BLV cases

**Figure 3:**
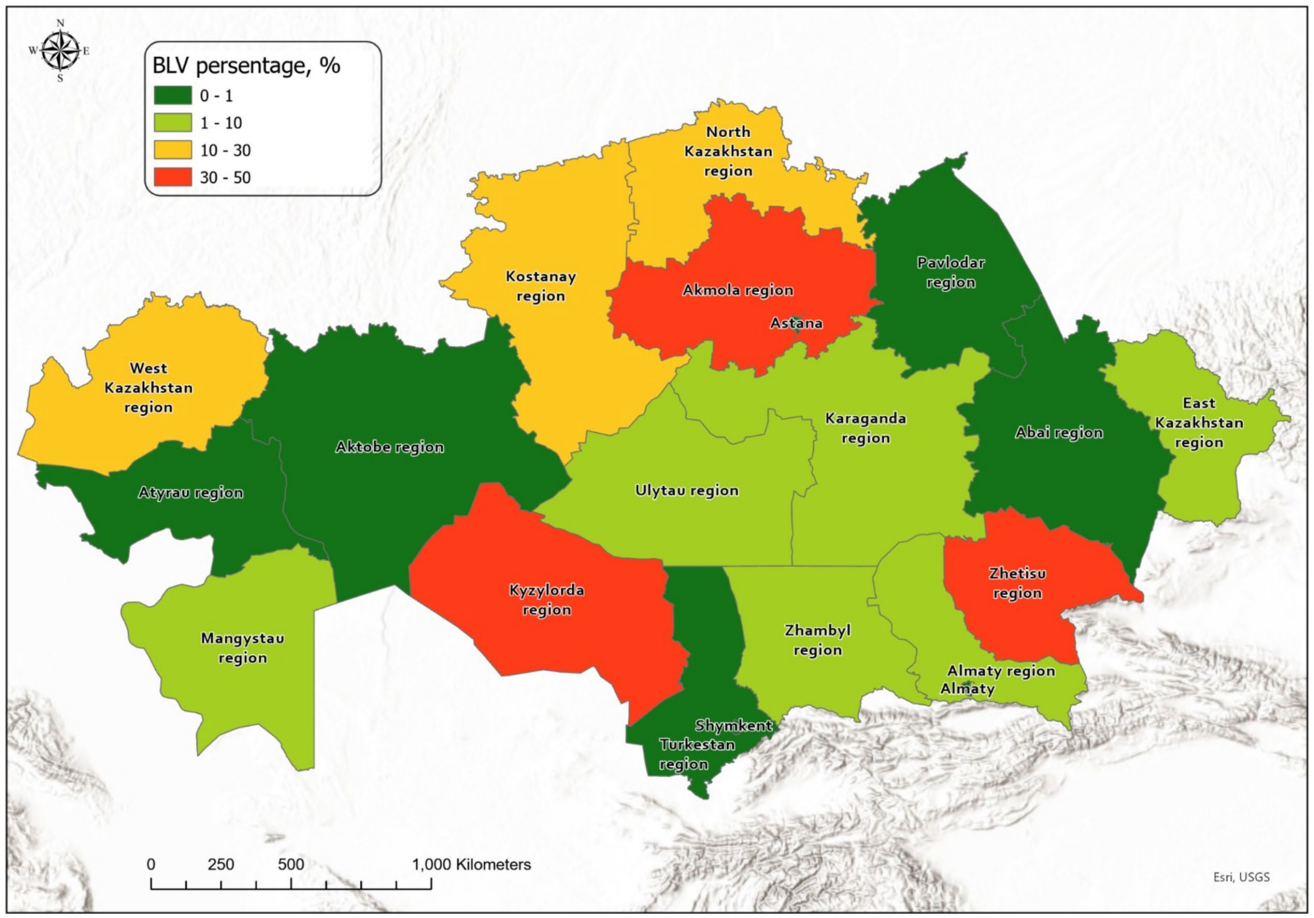
Administrative regions were classified into low-, moderate-, and high-risk zones according to predefined thresholds based on ELISA seroprevalence, with PCR used as confirmatory evidence of circulation (section 2.6).

GIS-based visualization revealed pronounced spatial heterogeneity in the distribution of BLV across Kazakhstan in 2025. Both seropositive and PCR-confirmed cases were unevenly distributed, forming localized clusters in specific administrative regions, while extensive territories remained epidemiologically stable throughout the surveillance period. Administrative regions were classified into low-, moderate-, and high-risk zones according to the prespecified criteria described in Section 2.6 (Figure 3).

This spatial zoning framework highlights areas requiring intensified surveillance and targeted control measures, as well as regions where routine monitoring was considered sufficient based on the 2025 data.

The spatial risk zoning demonstrates marked regional heterogeneity in BLV epidemiological status across Kazakhstan. High-risk zones were primarily concentrated in regions with consistently elevated seroprevalence and molecular confirmation of infection, whereas low-risk zones were characterized by the absence of both serological and PCR evidence of BLV circulation during the study period.

**Figure 4:**
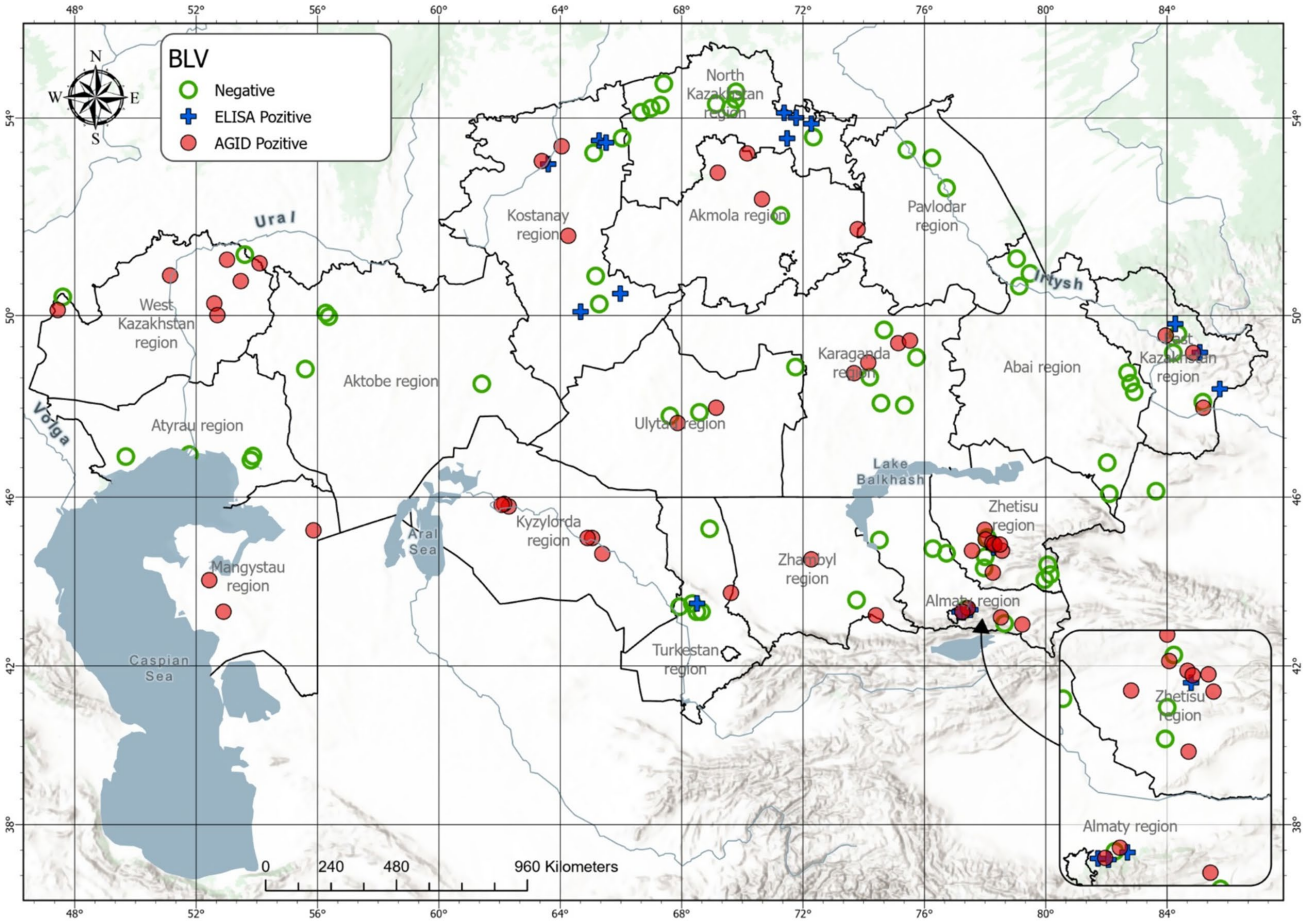
Integrated spatial risk zoning of BLV in Kazakhstan, 2025.

To visualize the spatial distribution of BLV infection across Kazakhstan, GIS-based mapping was performed using integrated serological (AGID and ELISA) and molecular (PCR) surveillance data obtained in 2025 (Figure 4).

GIS-based mapping illustrates the spatial distribution of BLV-negative animals (green), ELISA-positive cases (red), and AGID-positive cases (blue) across administrative regions. The map highlights pronounced spatial heterogeneity in BLV occurrence and demonstrates the co-circulation of serologically confirmed infections in regions with differing epidemiological profiles.

The coexistence of ELISA- and AGID-positive cases within the same regions reflects varying stages of infection detection and supports the complementary use of serological assays in large-scale surveillance.

## 4 Discussion

This study provides an integrated assessment of BLV epidemiology in Kazakhstan in 2025 through the combined application of serological testing, molecular detection, and spatial analysis. By integrating multiple diagnostic layers within a nationwide cross-sectional surveillance framework, the study offers a refined interpretation of BLV circulation in an endemic setting and highlights the practical value of diagnostic complementarity for epidemiological assessment.

BLV remains endemic in many cattle-producing regions and continues to pose challenges for surveillance because infection is frequently subclinical and detection depends on the performance characteristics and field implementation of serological and molecular assays. Previous studies have shown that serological screening is effective for population-level monitoring, whereas PCR-based detection provides complementary information on proviral DNA–positive animals and can support confirmation in selected cases. However, evidence from large-scale, geographically stratified surveillance in Central Asia remains limited, and most available datasets are fragmented by region, production system, or diagnostic approach. In Kazakhstan, recent nationwide BLV surveillance has confirmed endemicity and highlighted region-specific heterogeneity, supporting the need for integrated diagnostic strategies (13). In this context, our nationwide integrated dataset provides a consolidated view of BLV distribution in Kazakhstan and enables a direct comparison of assay outputs alongside spatial risk patterning. Overall, our findings are in agreement with previously published BLV surveillance data from Kazakhstan, while extending them through nationwide spatial stratification and integrated multi-layer diagnostics (13, 16).

A central finding of this work is the pronounced spatial heterogeneity of BLV circulation across Kazakhstan. Rather than a uniform national distribution, BLV detection was concentrated in specific regions, while other territories remained epidemiologically stable during the surveillance period. Such heterogeneity is consistent with the biological characteristics of BLV, whose transmission depends primarily on the transfer of infected lymphocytes and is therefore closely associated with management practices, animal movements, and iatrogenic factors rather than environmental persistence. These features underscore the limitations of interpreting national surveillance data without explicit spatial stratification. This pattern is consistent with previously reported Kazakhstan-wide observations that emphasized regional clustering of BLV positivity (13).

The comparison of serological and molecular diagnostic methods revealed overlapping but non-identical subsets of infected animals. Serological assays identified the majority of BLV-positive cattle, reflecting established immune responses, whereas PCR provided direct evidence of proviral DNA and enabled confirmation of infection at the molecular level. The detection of proviral DNA in a limited number of seronegative animals is consistent with early or atypical stages of infection and supports the complementary role of molecular testing within integrated surveillance programs. Importantly, discrepancies between AGID, ELISA, and PCR results observed in this study are best explained by differences in diagnostic sensitivity and infection stage rather than by methodological inconsistency. In this respect, our results align with integrated serological and molecular findings previously reported for Kazakhstan, supporting the interpretive value of combining assay outputs rather than relying on a single diagnostic modality (13, 15, 21).

Within the serological component of surveillance, AGID and ELISA fulfilled distinct but complementary functions. ELISA demonstrated higher analytical sensitivity and suitability for large-scale screening, particularly in regions with low to moderate BLV prevalence. AGID, despite its lower sensitivity, identified animals with established serological responses and remains operationally relevant in endemic settings where laboratory infrastructure, regulatory frameworks, and cost considerations influence diagnostic strategy. The concurrent use of both assays improved the interpretability of population-level data and strengthened confidence in regional prevalence estimates.

Molecular detection further enhanced epidemiological resolution by confirming infection at the genomic level in selected animals. Partial sequencing of PCR-positive samples verified BLV identity and demonstrated clustering within established BLV genotypes previously reported in the region. Although sequencing was performed on a limited subset of samples and was not intended to support comprehensive phylogenetic reconstruction, the observed genetic similarity to regional reference strains is consistent with known patterns of BLV circulation in geographically connected areas. These findings support the validity of molecular confirmation while avoiding overinterpretation of transmission pathways.

The integration of diagnostic results with GIS-based spatial analysis enabled classification of administrative territories into low-, moderate-, and high-risk zones. This risk-based zoning represents a key practical outcome of the study, translating heterogeneous diagnostic data into an interpretable framework for surveillance planning. In high-risk zones, intensified monitoring and targeted control measures may be warranted, whereas in low-risk areas, routine surveillance may be sufficient to maintain epidemiological stability. Such stratification aligns with contemporary principles of risk-based animal health management and resource allocation.

Several limitations should be acknowledged. The analysis was based on cross-sectional data from a single year and therefore does not capture temporal trends in BLV dynamics. Molecular testing and sequencing were applied to selected subsets of samples, which limits inference regarding viral diversity and genotype distribution. In addition, detailed herd-level management and movement data were not uniformly available, precluding formal analysis of specific transmission drivers. In addition, the surveillance-based sampling design (rather than formal random sampling) may limit strict generalization of prevalence estimates, although broad regional coverage mitigates this constraint.

In addition, spatial heterogeneity was interpreted based on GIS visualization; formal spatial statistics to quantify clustering (e.g., Moran’s I or hotspot/cluster detection) were beyond the scope of the present analysis and should be addressed in future work.

In conclusion, this study demonstrates that integrated surveillance combining serological testing, molecular detection, and spatial analysis provides a robust and informative framework for assessing BLV epidemiology in endemic settings. The findings from Kazakhstan in 2025 highlight the importance of diagnostic complementarity and spatial context for accurate interpretation of surveillance data and support the adoption of risk-based approaches to BLV monitoring and control in comparable epidemiological environments.

## Data Availability

The nucleotide sequences generated in this study have been deposited in the NCBI GenBank database under accession numbers PX367239–PX367242.
